# The systemic renin-angiotensin system in COVID-19

**DOI:** 10.1038/s41598-022-24628-1

**Published:** 2022-11-22

**Authors:** Roman Reindl-Schwaighofer, Sebastian Hödlmoser, Oliver Domenig, Katharina Krenn, Farsad Eskandary, Simon Krenn, Christian Schörgenhofer, Benedikt Rumpf, Mario Karolyi, Marianna T. Traugott, Agnes Abrahamowicz, Viktoria Tinhof, Hannah Mayfurth, Vincent Rathkolb, Sebastian Mußnig, Lukas Schmölz, Roman Ullrich, Andreas Heinzel, Franz König, Christina Binder, Diana Bonderman, Robert Strassl, Elisabeth Puchhammer-Stöckl, Gregor Gorkiewicz, Judith H. Aberle, Bernd Jilma, Christoph Wenisch, Marko Poglitsch, Rainer Oberbauer, Alexander Zoufaly, Manfred Hecking

**Affiliations:** 1 Clinical Division of Nephrology and Dialysis, Department of Internal Medicine III, Medical University of Vienna, Vienna, Austria; 2 Department of Epidemiology, Medical University Vienna, Vienna, Austria; 3 Attoquant Diagnostics, Vienna, Austria; 4 Department of Anesthesia, General Intensive Care and Pain Medicine, Medical University Vienna, Vienna, Austria; 5 Department of Clinical Pharmacology, Medical University Vienna, Vienna, Austria; 6 Department of Medicine IV, Klinik Favoriten, Vienna, Austria; 7 Department of Anesthesiology and Intensive Care Medicine, AUVA Trauma Centre Vienna, Vienna, Austria; 8 Center for Medical Statistics, Informatics and Intelligent Systems, Medical University Vienna, Vienna, Austria; 9 Department of Cardiology, Medical University Vienna, Vienna, Austria; 10 Department of Cardiology, Klinik Favoriten, Vienna, Austria; 11 Division of Virology, Department of Laboratory Medicine, Medical University Vienna, Vienna, Austria; 12 Center for Virology, Medical University Vienna, Vienna, Austria; 13 Institute of Pathology, Medical University of Graz, Graz, Austria; 14 Faculty of Medicine, Sigmund Freud University, Vienna, Austria

**Keywords:** Cardiovascular biology, Biomarkers, SARS-CoV-2

## Abstract

SARS-CoV-2 gains cell entry via angiotensin-converting enzyme (ACE) 2, a membrane-bound enzyme of the “alternative” (alt) renin-angiotensin system (RAS). ACE2 counteracts angiotensin II by converting it to potentially protective angiotensin 1–7. Using mass spectrometry, we assessed key metabolites of the classical RAS (angiotensins I–II) and alt-RAS (angiotensins 1–7 and 1–5) pathways as well as ACE and ACE2 concentrations in 159 patients hospitalized with COVID-19, stratified by disease severity (severe, n = 76; non-severe: n = 83). Plasma renin activity (PRA-S) was calculated as the sum of RAS metabolites. We estimated ACE activity using the angiotensin II:I ratio (ACE-S) and estimated systemic alt-RAS activation using the ratio of alt-RAS axis metabolites to PRA-S (ALT-S). We applied mixed linear models to assess how PRA-S and ACE/ACE2 concentrations affected ALT-S, ACE-S, and angiotensins II and 1-7. Median angiotensin I and II levels were higher with severe versus non-severe COVID-19 (angiotensin I: 86 versus 30 pmol/L, *p* < 0.01; angiotensin II: 114 versus 58 pmol/L, *p* < 0.05), demonstrating activation of classical RAS. The difference disappeared with analysis limited to patients not taking a RAS inhibitor (angiotensin I: 40 versus 31 pmol/L, *p* = 0.251; angiotensin II: 76 versus 99 pmol/L, *p* = 0.833). ALT-S in severe COVID-19 increased with time (days 1–6: 0.12; days 11–16: 0.22) and correlated with ACE2 concentration (r = 0.831). ACE-S was lower in severe versus non-severe COVID-19 (1.6 versus 2.6; *p* < 0.001), but ACE concentrations were similar between groups and correlated weakly with ACE-S (r = 0.232). ACE2 and ACE-S trajectories in severe COVID-19, however, did not differ between survivors and non-survivors. Overall RAS alteration in severe COVID-19 resembled severity of disease-matched patients with influenza. In mixed linear models, renin activity most strongly predicted angiotensin II and 1-7 levels. ACE2 also predicted angiotensin 1-7 levels and ALT-S. No single factor or the combined model, however, could fully explain ACE-S. ACE2 and ACE-S trajectories in severe COVID-19 did not differ between survivors and non-survivors. In conclusion, angiotensin II was elevated in severe COVID-19 but was markedly influenced by RAS inhibitors and driven by overall RAS activation. ACE-S was significantly lower with severe COVID-19 and did not correlate with ACE concentrations. A shift to the alt-RAS axis because of increased ACE2 could partially explain the relative reduction in angiotensin II levels.

## Introduction

The renin-angiotensin system (RAS) is a complex network that regulates blood pressure and blood volume through the hormone angiotensin II via its type 1 (AT_1_) receptor (‘classical’ RAS)^1^. Angiotensin 1-7 acts via an ‘alternative’ RAS pathway. It partially antagonizes AT_1_ receptor–mediated effects such as vasoconstriction, fibrosis, and thrombogenesis by signaling through the G-protein–coupled Mas receptor, which promotes vasodilation and anti-fibrotic actions^2^. Although angiotensin-converting enzyme (ACE), a carboxypeptidase, drives angiotensin II formation from angiotensin I, ACE2 opposes its action through conversion of angiotensin II to angiotensin 1-7^3^.

Severe Acute Respiratory Syndrome (SARS)–Coronavirus (CoV)-2 uses ACE2 as its primary entry receptor into human cells^4^. ACE2 is a membrane-anchored protein expressed on the surface of type II alveolar cells and epithelial cells of the lung. It also is present in many other tissues, including the intestine, kidney, and heart, and can be shed following cleavage by ADAM17^5^. COVID-19, the disease caused by SARS-CoV-2, has a highly variable clinical presentation and severity, with age, male sex, and comorbidities such as diabetes and hypertension associated with increased severity risk^6^.

The assumption that the RAS becomes dysregulated in COVID-19 is based on experimental data showing that ACE2 is downregulated in the lungs of wild-type mice infected with SARS-CoV^7^. This downregulation was inferred to contribute to lung damage by impairing angiotensin II degradation^7,8^, and systemic angiotensin II infusion in a large animal model led to lung injury closely resembling COVID-19^9^. Furthermore, in one hospital study, angiotensin II levels were elevated in 12 patients with COVID-19 compared with eight healthy controls, and were associated with a higher SARS-CoV-2 viral load and lower PaO_2_/FiO_2_ ratio as a read-out for lung injury^10^.

We recently reported that endogenous enzymatically active ACE2 is increased in severe COVID-19 to concentrations that may significantly affect systemic RAS regulation^11^. In the present study, we compared changes in classical and alternative RAS regulation between patients with severe versus non-severe COVID-19.

## Methods

### Study cohort and sampling

The study cohort comprises patients with PCR-confirmed SARS-CoV-2 infection who were hospitalized for COVID-19 at Klinik Favoriten in Vienna, Austria, from March 15 to September 30, 2020, and enrolled in the Austrian COronaVirus Adaptive Clinical Trial (ACOVACT, registered with clinicaltrials.gov [NCT04351724]. This study was approved by the Ethics Committee of the Medical University of Vienna [#1315/2020]). ACOVACT is a multicenter, randomized, active-controlled, open-label platform trial to evaluate the efficacy and safety of therapeutics for COVID-19. Serum or plasma was sampled up to three times weekly in hospitalized patients. Patients provided written informed consent to participate. To place the RAS regulation status of Covid-19 patients into context with other states of health and disease, we measured RAS profiles in 16 healthy individuals and re-analyzed RAS data from 27 critically ill patients with influenza pneumonia included in a previous study, serving as controls^11^.

### Data extraction and assessment of clinical status

Data extraction was done by chart review. RAS medication status was defined as treatment with a pharmacological RAS inhibitor at the time of hospitalization, confirmed by direct measurement of drug concentration in the circulation. COVID-19 severity was defined by the maximum requirement for respiratory support during hospitalization and categorized as severe (invasive and non-invasive ventilation, including high-flow nasal cannula oxygen therapy; WHO scale 6–10) and non-severe (all other hospitalized patients; WHO scale 4,5)^12^. Clinical co-variables included age, sex, BMI, as well as history of hypertension, diabetes, and chronic obstructive pulmonary disease. All have previously been described as risk factors for severe COVID-19^13,14^.

### Angiotensin quantification (RAS-Fingerprint)

The equilibrium levels of six angiotensin peptide metabolites (angiotensins I–IV, angiotensin 1-7, angiotensin 1–5) in human heparinized plasma samples were quantified by liquid chromatography-mass spectrometry/mass-spectroscopy (LC–MS/MS) using previously described methods^15,16^. The biochemical background of the equilibrium approach has recently been validated^17,18^. To calculate overall RAS activity, we used the sum of RAS metabolites (angiotensins I-IV, 1-7, and 1–5) to yield a plasma renin activity (PRA-S) value. To estimate ACE activity, we took the ratio of angiotensins I and II, yielding an activity marker for ACE, the ACE-S. For an estimate of systemic alternative RAS pathway activity (ALT-S), we calculated the ratio of the sum of alternative RAS metabolites (angiotensins 1–7 and 1–5) and the PRA-S value as previously described^19,20^.

### Measurement of ACE and ACE2

ACE and ACE2 concentrations were determined in human heparinized or citrate plasma (diluted in substrate/product stabilizing buffer; ACE: 10 µM MLN-4760, 10 µM Aminopeptidase Inhibitor, 20 µM Z-Pro-Prolinal, 1 mM ZnCl_2_; ACE2: 10 µM Lisinopril, 10 µM Aminopeptidase inhibitor, 20 µM Z-Pro-Prolinal, 1 mM ZnCl_2_) after samples were spiked with their respective substrates (angiotensin I for ACE and angiotensin II for ACE2) and incubated at 37 °C with or without specific inhibitors (ACE: 10 µM lisinopril; ACE2: 10 µM ML N-4760). We used LC–MS/MS to quantify the obtained products (angiotensin II from ACE activity; angiotensin 1-7 from ACE2 activity). To calculate the enzyme-specific angiotensin formation rate and determine active ACE and ACE2 concentrations, we used a calibration curve of recombinant human ACE (R&D Systems, Minneapolis, MN, USA) over ACE2 in human serum or plasma.

### Measurement of RAS inhibitor medications

Following methanol precipitation, samples were diluted in injection buffer (10% acetonitrile/0.1% formic acid) and subjected to mass spectrometry analysis using a reversed analytical column (Acquity UPLC C18, Waters) operating in line with a XEVO TQ-S triple quadrupole mass spectrometer (Waters Xevo TQ/S, Milford, MA, USA) in multiple reaction monitoring mode. Drug concentrations (candesartan, valsartan, losartan, olmesartan, telmisartan, enalaprilat, lisinopril, perindoprilat, ramiprilat) were calculated from integrated chromatograms, considering the corresponding response factors determined in appropriate calibration curves for the serum matrix when integrated signals exceeded a signal-to-noise ratio of 10.

### Ex vivo assessment of ALT-S at different ACE2 levels

The alternative RAS (ALT-S) was investigated in in pooled serum samples following addition of different concentrations of recombinant human ACE2 (rhACE2).

### Statistical analysis

Patient characteristics are described by medians and first and third quartiles for continuous variables and frequencies and percentages for categorical variables. Groups were compared using Student’s t-test for continuous variables with normal distribution and chi-squared and Fisher’s exact tests for categorical variables. The starting point for all analyses over time was the day of hospitalization for SARS-CoV-2. Because of multiple measurements per patient, to compare changes over time, we calculated mean angiotensin levels during the hospital stay and over 5-day time intervals. Values for biomarkers were largely not normally distributed, so we used Mann–Whitney U tests to compare angiotensin levels between severity groups (severe versus non-severe). To visualize selected angiotensin levels over time, we used non-parametric smoothing (i.e., the *loess* function in R) in conjunction with local 95% confidence intervals. In addition, we employed boxplots to visualize ACE2 concentrations and angiotensin levels in the two disease severity groups and across 5-day intervals. We generated scatterplots of selected angiotensin pairs (on a log axis where necessary), color-coded by disease severity or ACE2 value (on a logarithmic color scale), and reported Spearman correlation coefficients.

To evaluate associations of levels of the RAS-Fingerprint components (ACE-S, ALT-S, angiotensin 1-7, angiotensin II) with other variables, we fitted linear mixed effects models with random intercept per patient, with varying independent variables (separate models using PRA-S, ACE, and ACE2, and a combined model with all three as covariates). Dependent variables as well as covariates were log transformed. Models were compared using pseudo R^2^ for generalized mixed effects models, specifically by the marginal R^2^, denoted by ${R}_{m}^{2}$^21^ This type of statistic represents the variance explained by the fixed effects, so we interpret it as a goodness-of-fit indicator at the population level. We considered two-sided *p* < 0.05 as indicating statistical significance. Because we did not test a specific hypothesis and used statistical tests purely for descriptive purposes, we did not adjust p values for multiple testing. All calculations and statistical analyses were performed using R 4.0.1.

### Ethical approval and consent to participate

The Austrian COronaVirus Adaptive Clinical Trial (ACOVACT) was approved by the Ethics Committee of the Medical University of Vienna (#1315/2020) and registered with clinicaltrials.gov (NCT04351724). All participating individuals were adults and provided their written, informed consent to participate. The study was conducted in accordance with the principles of the Declaration of Helsinki.

## Results

### Characteristics of the study patients and clinical outcomes

**Figure 1:**
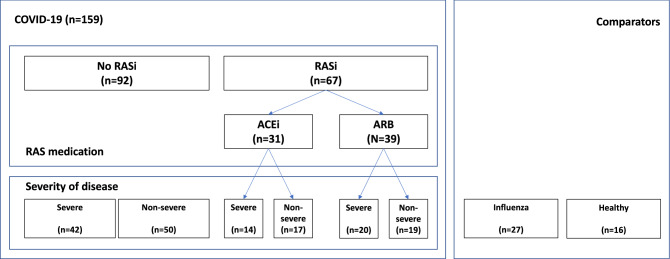
Study cohort.

A total of 159 patients, all hospitalized with COVID-19, were included in the study (Fig. 1). Baseline characteristics and clinical parameters of the patients, stratified by COVID-19 severity (severe versus non-severe), are provided in Table 1. The median age across the whole group was 66 years, and 32.9% were women, with no significant differences between disease severity groups. Of the entire study cohort, 19.6% and 24.7% of patients received an ACE inhibitor (ACEi) or an angiotensin receptor blocker (ARB) at baseline and during the study, respectively.

**Table 1:** Demographic and clinical characteristics of the study cohort.

	Non-severe (n = 83)	Severe (n = 76)	Total (N = 159)	*P*
Age	67.0 [52.5, 79.3]	64.5 [52.3, 73.0]	65.5 [52.3, 76.8]	0.613
Female	31 (39.2%)	19 (26.0%)	50 (32.9%)	0.057
BMI	27.7 [24.8, 30.6]	25.8 [24.5, 30.7]	26.5 [24.5, 30.8]	0.947
ACEi	17 (20.5%)	14 (18.7%)	31 (19.6%)	0.774
ARB	20 (24.1%)	19 (25.3%)	39 (24.7%)	0.857
Diabetes	22 (26.5%)	24 (32.0%)	46 (29.1%)	0.448
Hypertension	49 (59.0%)	49 (65.3%)	98 (62.0%)	0.415
COPD	5 (6.0%)	12 (16.0%)	17 (10.8%)	0.043
Hospital length of stay (days)	14.0 [10.0, 25.0]	28.0 [16.8, 33.3]	21.0 [12.0, 30.0]	< 0.001
Death	2 (2.4%)	18 (23.7%)	20 (12.6%)	< 0.001

Continuous variables are presented as medians [with first, third quartiles), and binary variables are presented as absolute numbers (with %).

Median in-hospital length of stay was 28 days for those with severe disease and 14 days for those with non-severe COVID-19. All patients were followed until discharge or death. A total of 15 patients died, corresponding to a mortality rate of 23.7% with severe disease versus 2.4% with non-severe COVID-19.

### Overall RAS activity

**Figure 2:**
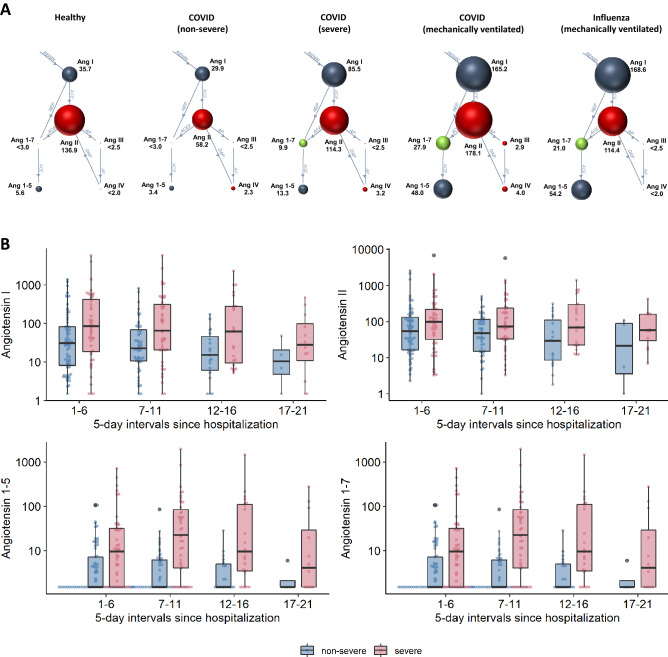
RAS metabolite levels. (**A**) Classical and alternative RAS metabolite levels in healthy individuals (n = 16), COVID-19 patients of different severity (non-severe [n = 83], severe [n = 76]; subgroup of mechanically ventilated [n = 44] as well as patients with severe influenza pneumonia [n = 27]; Diameter of the spheres represent median levels of angiotensins, values are provided on the side. (**B**) Angiotensin levels stratified for severe and non-severe COVID-19 and time after hospitalization (5-day intervals); all angiotensin concentrations in pmol/L.

**Figure 3:**
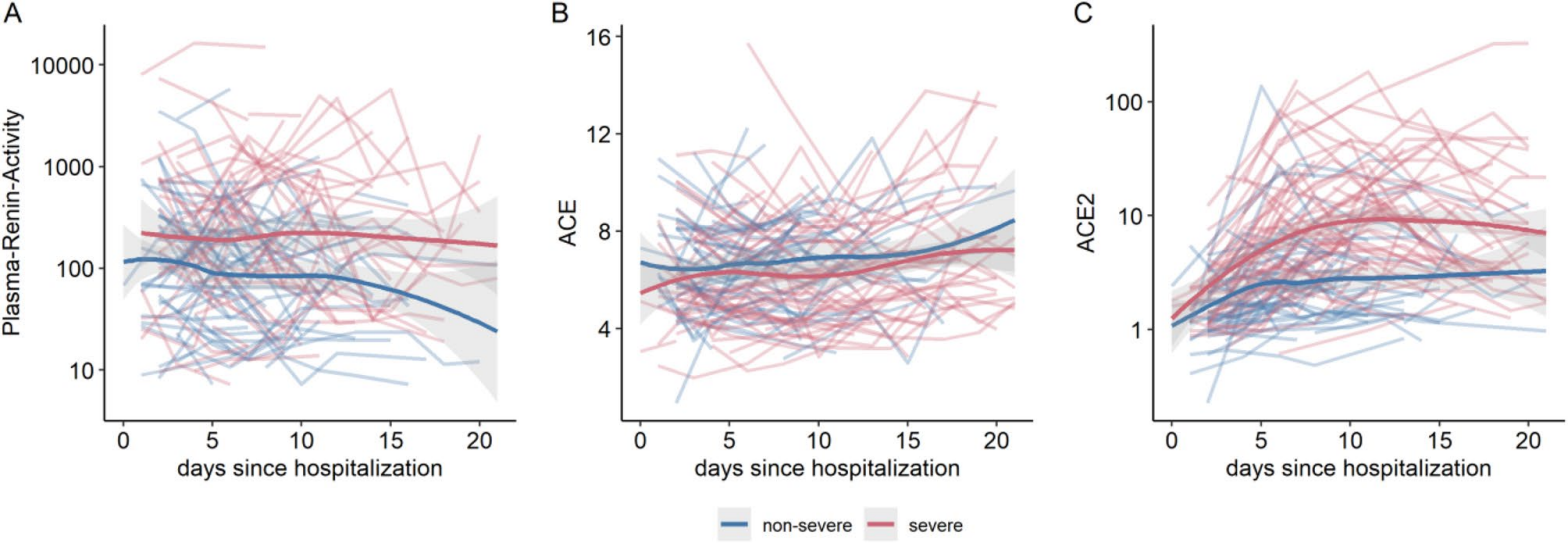
Key RAS enzymes. Key RRAS enzymes over time stratified for severe and non-severe COVID-19. PRA-S is reported as a unitless ratio; ACE and ACE 2 are reported in µg/mL and ng/mL, respectively.

A total of 680 samples were available for RAS analysis (complete RAS profiles were available for 511 samples; RAS enzyme quantification alone was performed for the remaining 169 samples). Median systemic angiotensin levels stratified by disease severity and across 5-day intervals following hospitalization are reported in Tables 2A,B. In severe versus non-severe COVID-19, we observed an overall RAS activation with higher angiotensin I and angiotensin II levels (86 versus 30 pmol/L angiotensin I, *p* < 0.01; 114 versus 58 pmol/L angiotensin II, *p* < 0.05; Fig. 2A,B). In line with this pattern, PRA-S was also elevated in severe compared with non-severe COVID-19 (Fig. 3A). Overall, the highest angiotensin I and II levels overall were observed in invasively ventilated, critically ill patients (n = 44; 271 pmol/L angiotensin I and 207 pmol/L angiotensin II, Table S3).

**Table 2:** RAS analysis in patients with COVID-19.

	All patients			No ACEi/ARB			ACEi			ARB		
	Non-severe	Severe	P	Non-severe	Severe	P	Non-severe	Severe	P	Non-severe	Severe	P
*(2A) Across all time points*												
Angiotensin I	29.91 [9.03, 72.54]	85.50 [26.00, 321.62]	< 0.01*	30.66 [7.29, 64.39]	40.19 [10.20, 232.31]	0.251	70.61 (10.87,346.86)	315.25 (123.74,466.38)	0.078	14.40 (8.63,46.96)	110.56 (58.10,213.68)	< 0.05*
Angiotensin II	58.17 [16.83, 131.78]	114.30 [52.16, 236.14]	< 0.05*	98.57 [25.66, 169.77]	76.28 [31.55, 215.08]	0.833	16.86 (6.07,34.96)	144.13 (107.61,272.54)	< 0.01*	62.17 (28.47,101.28)	159.10 (87.15,255.95)	0.072
Angiotensin 1–5	3.37 [1.76, 8.70]	13.26 [5.01, 48.55]	< 0.001*	4.06 [1.97, 9.49]	10.22 [2.71, 45.16]	0.051	1.93 (1.00,3.16)	16.20 (10.38,20.51)	< 0.01*	3.87 (2.46,7.95)	20.56 (4.57,50.89)	0.071
Angiotensin 1-7	1.97 [1.50, 6.84]	9.92 [2.80, 38.03]	< 0.001*	1.93 [1.50, 5.29]	7.11 [1.50, 31.28]	< 0.05*	2.40 (1.50,12.92)	19.60 (10.77,31.31)	< 0.05*	1.50 (1.50,5.97)	10.36 (5.80,41.58)	< 0.05*
PRA-S	130.75 [39.44, 254.74]	293.80 [129.67, 681.86]	< 0.05*	148.38 [39.47, 250.81]	139.31 [45.50, 473.49]	0.509	89.93 (39.36,434.57)	402.99 (293.80,824.41)	< 0.05*	85.91 (46.50,164.30)	355.59 (228.89,561.14)	< 0.05*
ACE	6.66 [5.01, 7.65]	6.09 [4.85, 7.96]	0.636	6.69 [5.14, 8.01]	5.50 [4.69, 7.73]	0.197	5.81 (4.97,7.37)	8.08 (7.44,9.11)	< 0.05*	6.07 (4.68,7.40)	5.89 (5.39,6.15)	0.405
ACE2	1.84 [1.27, 2.83]	5.71 [3.15, 16.14]	< 0.001*	1.80 [1.23, 2.70]	4.81 [3.18, 14.02]	< 0.001*	1.80 (1.39,4.14)	4.45 (3.39,10.70)	< 0.01*	2.33 (1.39,2.76)	6.94 (3.08,20.64)	< 0.01*
ALT-S	0.07 [0.05, 0.11]	0.12 [0.08, 0.21]	< 0.001*	0.07 [0.05, 0.12]	0.13 [0.09, 0.21]	< 0.001*	0.06 (0.05,0.09)	0.11 (0.09,0.11)	0.17	0.08 (0.07,0.11)	0.12 (0.07,0.21)	0.128
ACE-S	2.63 [1.89, 3.62]	1.60 [0.83, 2.06]	< 0.001*	2.94 [2.24, 3.82]	1.77 [0.90, 2.32]	< 0.001*	0.42 (0.07,1.68)	0.79 (0.70,1.17)	0.183	2.60 (2.11,3.33)	1.51 (1.37,2.08)	< 0.001*

	Interval (days)	Non-severe	Severe	*P*								
*(2B) At 5-day time intervals*												
Angiotensin I	(1–6)	30.59 [8.16, 81.77]	84.29 [18.74, 424.07]	< 0.05*								
	(6–11)	22.70 [10.58, 68.61]	65.12 [20.49, 317.57]	< 0.01*								
	(11–16)	15.05 [6.08, 46.61]	61.03 [9.56, 278.48]	0.052								
	(16–21)	11.20 [5.60, 23.38]	28.14 [13.13, 103.13]	0.248								
Angiotensin II	(1–6)	54.04 [16.25, 132.99]	98.69 [32.89, 217.90]	0.091								
	(6–11)	47.76 [15.03, 115.09]	72.82 [33.00, 235.84]	< 0.05*								
	(11–16)	29.15 [8.57, 113.15]	69.05 [22.42, 296.77]	< 0.05*								
	(16–21)	44.09 [4.36, 89.32]	57.43 [30.38, 159.41]	0.379								
Angiotensin 1-7	(1–6)	1.50 [1.50, 7.26]	9.69 [1.50, 31.66]	< 0.001*								
	(6–11)	1.50 [1.50, 6.16]	22.47 [4.07, 85.19]	< 0.001*								
	(11–16)	1.50 [1.50, 4.99]	9.56 [3.45, 109.93]	< 0.01*								
	(16–21)	1.50 [1.50, 2.61]	4.16 [1.50, 38.03]	0.244								
Angiotensin 1–5	(1–6)	3.22 [1.00, 7.07]	7.98 [2.57, 20.69]	< 0.01*								
	(6–11)	3.92 [2.02, 6.60]	22.84 [3.36, 80.96]	< 0.001*								
	(11–16)	3.91 [1.00, 9.06]	18.43 [8.07, 119.79]	< 0.001*								
	(16–21)	2.75 [1.00, 5.42]	10.97 [6.44, 63.49]	0.059								
PRA-S	(1–6)	112.19 [39.15, 285.18]	238.57 [76.23, 586.81]	< 0.05*								
	(6–11)	108.94 [39.11, 230.67]	253.36 [67.12, 928.85]	< 0.01*								
	(11–16)	54.79 [21.96, 203.97]	171.69 [46.85, 918.76]	< 0.05*								
	(16–21)	60.09 [12.47, 124.74]	136.02 [67.82, 340.22]	0.17								
ACE	(1–6)	6.45 [4.95, 7.76]	5.70 [4.75, 7.52]	0.664								
	(6–11)	6.98 [5.19, 8.05]	5.44 [4.55, 7.70]	0.073								
	(11–16)	6.11 [5.15, 8.17]	6.61 [4.66, 7.94]	0.852								
	(16–21)	7.15 [5.68, 9.68]	6.94 [5.79, 9.58]	0.909								
ACE2	(1–6)	1.75 [1.20, 3.02]	3.89 [1.77, 6.79]	< 0.001*								
	(6–11)	2.41 [1.46, 4.06]	7.58 [4.15, 19.83]	< 0.001*								
	(11–16)	1.77 [1.27, 5.45]	9.26 [4.00, 20.28]	< 0.001*								
	(16–21)	2.26 [1.51, 3.75]	5.78 [2.81, 18.35]	0.051								
ALT-S	(1–6)	0.07 [0.04, 0.11]	0.10 [0.06, 0.17]	< 0.01*								
	(6–11)	0.08 [0.05, 0.13]	0.17 [0.12, 0.28]	< 0.001*								
	(11–16)	0.12 [0.07, 0.14]	0.21 [0.15, 0.30]	< 0.001*								
	(16–21)	0.14 [0.07, 0.20]	0.16 [0.09, 0.39]	0.446								
ACE-S	(1–6)	2.66 [1.70, 3.67]	1.51 [0.86, 1.95]	< 0.001*								
	(6–11)	2.60 [1.76, 3.41]	1.27 [0.76, 1.84]	< 0.001*								
	(11–16)	3.01 [1.80, 4.09]	1.84 [1.04, 2.56]	0.079								
	(16–21)	3.84 [3.07, 4.60]	2.31 [1.11, 3.32]	0.485								

Values are presented as medians [with first, third quartiles]. Group comparisons (*p* values) were obtained using the Wilcoxon signed-rank test. All angiotensin concentrations, including PRA-S, are reported as pmol/L. ACE concentration is reported in µg/mL and ACE2 enzyme concentration in ng/mL. The angiotensin II:I ratio and the alternative RAS ratio wre calculated using peptide levels in pmol/l and are reported as unitless values.

We also assessed RAS profiles in the subgroup of 92 patients not receiving RAS inhibitors (Table S1) and found that the difference in classical RAS activation by severity had vanished (severe versus non-severe COVID-19, respectively: 40 versus 31 pmol/L angiotensin I, *p* = 0.251; and 76 versus 99 pmol/L angiotensin II, *p* = 0.833; Table 2A, Table S2). Conversely, activation of the classical RAS at time of hospitalization was highest in severe COVID-19 patients on RASi treatment (Tables 2A and S4). In line to the drugs mechanism of action, patients on ACEi treatment at time of hospitalization had higher angiotensin I levels, while patients on ARB showed higher angiotensin II levels.

RAS profiles in healthy individuals differed from COVID-19 showing lower Angiotensin I but higher Angiotensin II levels (Table S5). Angiotensin levels in mechanically ventilated influenza patients were comparable to severity of disease matched COVID-19 patients requiring mechanical ventilation (Table S6; median Sequential Organ Failure Assessment [SOFA] score of 9 ^8–11^ and 9 ^8–11^ for mechanically ventilated COVID-19 and influenza patients).

### Alternative RAS

**Figure 4:**
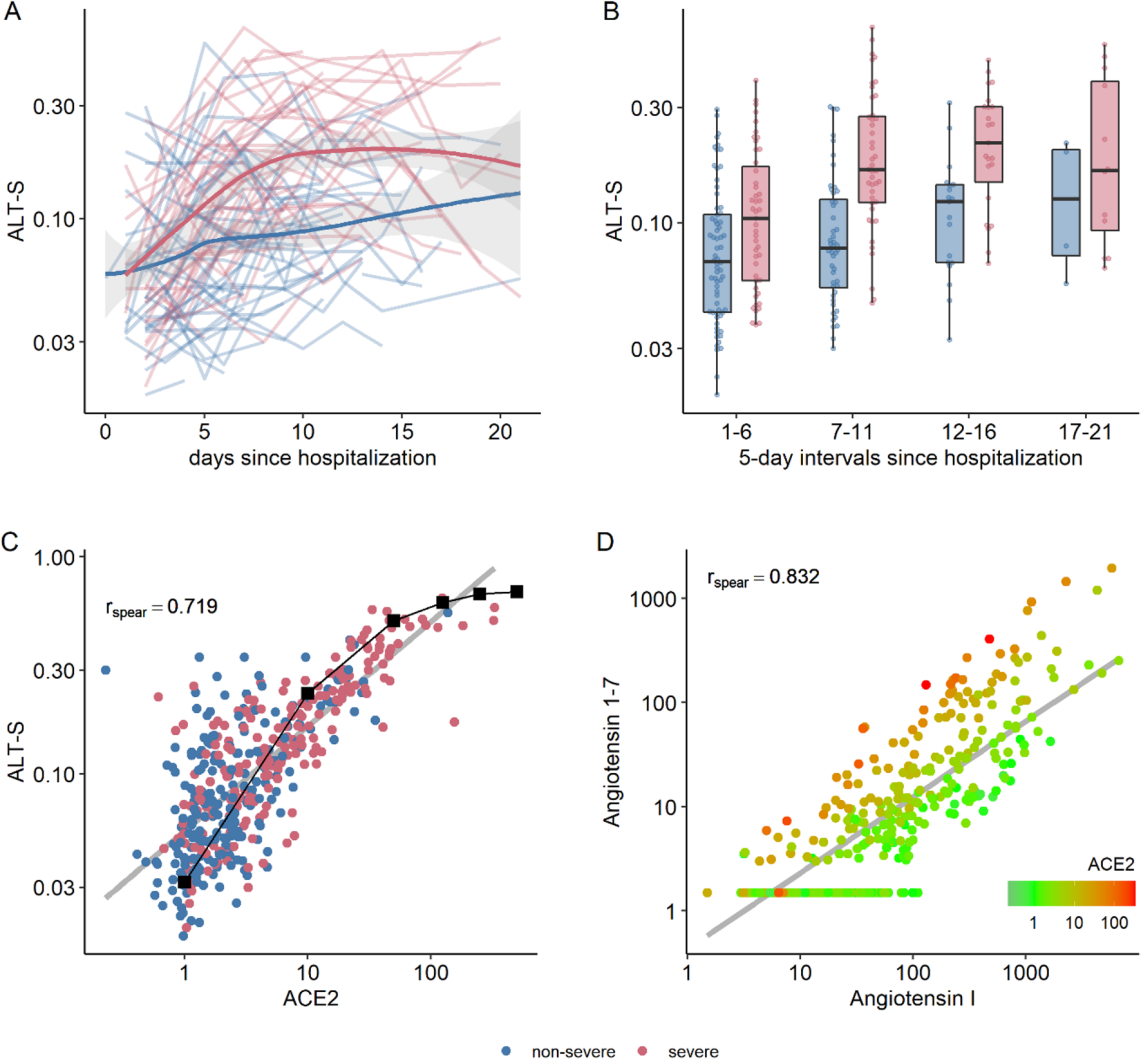
Alternative RAS. (**A**, **B**) The alternative RAS ratio (ALT-S) increased in severe COVID-19 compared with less severe cases over time. (**C**) The alternative RAS ratio was highly correlated with ACE2 levels in the circulation. There was a non-linear correlation between ACE2 and the alternative RAS-ratio in ex vivo experiments using pooled serum samples containing defined amounts of recombinant human ACE2 and renin (black squares, C). (**D**) Angiotensin 1-7 and angiotensin II (product and substrate of ACE2) showed a strong correlation that was modified by systemic ACE2 concentration.

In contrast to the pattern for overall RAS activity, we observed a marked increase in angiotensin 1-7 and angiotensin 1–5 levels in severe COVID-19, in line with an overall shift to the alternative RAS, independent of RAS inhibitor use (Table 2A,B, Figs. 2A,B and 4A,B). The increase in the alternative RAS component angiotensin 1-7 (and its downstream metabolite angiotensin 1–5) was highly correlated with systemic ACE2 concentrations (r = 0.831; Figs. 3C and 4C). The ALT-S (as ratio of alterative RAS metabolites on the total RAS activity) in severe COVID-19 increased from 10% during days 1–6 to 21% during days 11–16 of hospitalization (showing a shift to alternative RAS metabolites). Overall, angiotensin I and angiotensin 1-7 levels were highly correlated (r = 0.827), but ACE2 modified this relationship, and angiotensin 1-7 levels were relatively higher with higher ACE2 concentrations (Fig. 4D). Alternative RAS activation findings were similar in the subgroup of patients not taking RAS inhibitors (Table 2A).

Of interest, ALT-S plateaued at ACE2 levels > 100 ng/mL (Fig. 4C). We observed a similar plateau in an in vitro experiment using different concentrations of recombinant ACE2 in pooled plasma samples. These findings suggest that a limited availability of angiotensin II prevented production of higher levels of angiotensin 1-7 and angiotensin 1–5 (black squares, Fig. 4C).

ALT-S in healthy individuals was 0.04, while ALT-S was elevated in critically ill influence patients but overall lower compared to ALT-S in mechanically ventilated COVID-19 patients (0.14 and 0.24 for critically-ill influenza and COVID-19, respectively; Tables S5 and S6). ALT-S was highly correlated with ACE2 in patients with influenza (r = 0.821).

Importantly, median ACE2 levels did not differ between individuals taking ACEi or ARB and those not on RASi treatment (ARB: 2.83 ng/ml; ACEi 3.86 ng/ml; and not on RASi: 2.44 ng/ml; Table S7).

### ACE-S

**Figure 5:**
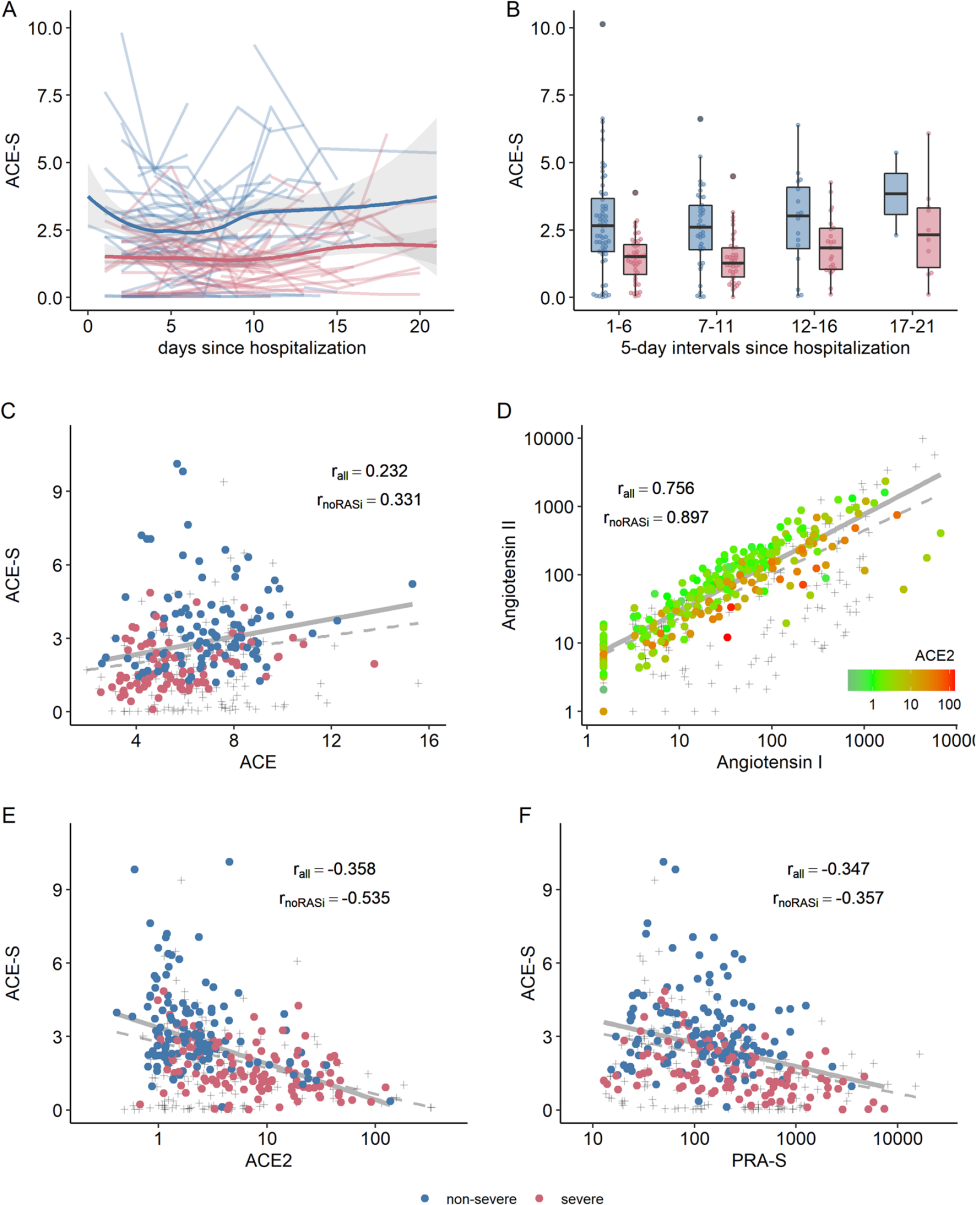
Ratio of angiotensin II to angiotensin I (**A**, **B**) The ratio of angiotensin II to angiotensin I (ALT-S) was reduced in severe COVID-19 throughout hospitalization. (**C**) ACE-S showed only a poor correlation with ACE concentration in the circulation. (**D**) Angiotensin II and angiotensin I showed a high correlation that was modified by systemic ACE2 concentration, resulting in a relative decrease of angiotensin II in relation to angiotensin I. (**E**,**F**) Both RAS activation (assessed by PRA-S, angiotensin-based plasma renin activity) and ACE2 showed moderate overall correlation with intrinsic ACE activity.

The systemic activity of ACE, estimated by the angiotensin-II-to-angiotensin-I ratio (ACE-S), was markedly lower with severe versus non-severe COVID-19 (1.6 vs. 2.6), suggesting decreased functional ACE activity (Fig. 5A,B). ACE concentration, however, was similar between the two groups (Table 2A,B and Fig. 3B), and the correlation between ACE-S and ACE concentration was weak (r = 0.232). When we excluded patients on RAS inhibitors, the correlation increased but remained rather weak (r = 0.331; Fig. 5C). The possibility of unaccounted use of RAS inhibitors as a potential confounder in individuals with low intrinsic ACE activity (< 2) was ruled out by direct measurement in patient sera for most RAS inhibitors (ACE inhibitors: enalaprilat, lisinopril, perindoprilat, ramiprilat; angiotensin receptor blockers: candesartan, valsartan, losartan, olmesartan, telmisartan).

We explored potential factors contributing to the reduced intrinsic ACE activity in severe COVID-19 with and without use of RAS inhibitors. Figure 5D shows the correlation between angiotensin I and angiotensin II, and thus ACE-S values, with lower values lying below the regression line and higher values above it. Overall, angiotensin I and II correlated strongly across all stages of overall RAS activity (r = 0.756 for all patients and r = 0.897 for patients not taking RAS inhibitors). For samples with values below the regression line (i.e., with lower ACE-S), however, we observed higher ACE2 levels. In line with this pattern, ACE2 levels showed a moderate negative correlation with ACE-S (r =  − 0.358 for all patients and − 0.535 for patients not taking RAS inhibitors; Fig. 5E). Furthermore, PRA-S showed a moderate negative correlation with ACE-S (r =  − 0.347 for all patients and r =  − 0.357 for patients not taking RAS inhibitors; Fig. 5F).

ACE-S in healthy individuals was 4.7 and thus higher compared to COVID-19 patients (Table S5). In patients with severe influenza, however, ACE-S was also decrease and comparable to patients with severe COVID-19 (1.49 and 1.51, respectively; Tables S6).

### Explanatory models

**Figure 6:**
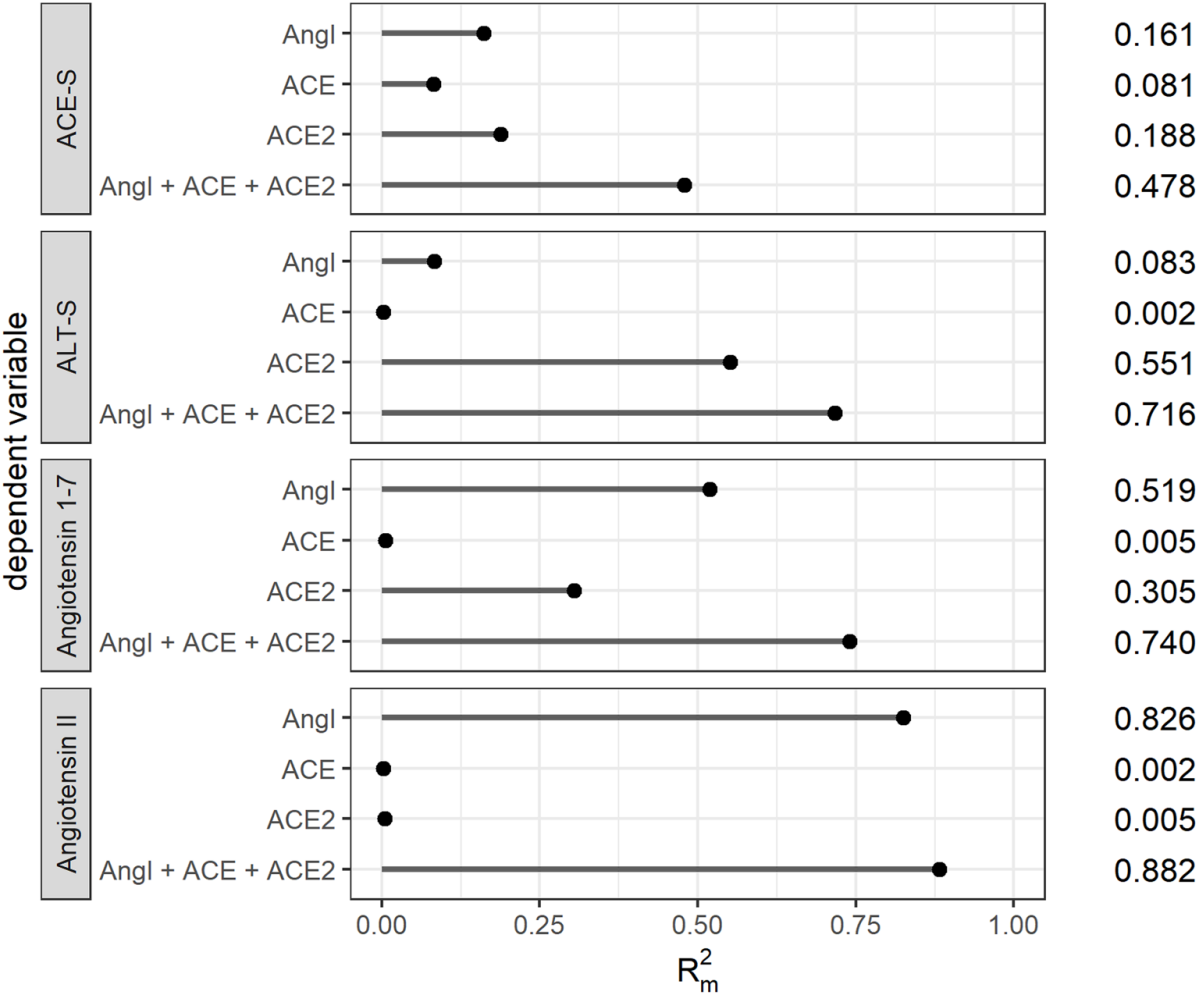
Mixed effects models. ${R}_{m}^{2}$, i.e., marginal R squared for linear mixed effects regression, modeling angiotension 1-7, angiotension II, the angiotension II:I ratio (ACE-S), and alternative RAS ratio (ALT-S); all dependent variables were modeled using enzyme plasma renin activity (PRA-S) and enzyme concentration of ACE and ACE2 as single covariates, as well as with a combined model. All models included a random intercept per patient. ${R}_{m}^{2}$ denotes the variance explained by the fixed effects of the respective model.

We subsequently applied mixed models to analyze the influence of RAS activity (assessed by PRA-S and concentrations of ACE and ACE2) on angiotensin II and angiotensin 1-7 levels as well as on ACE-S and ALT-S in patients without RAS inhibitory medication (Fig. 6, data for all patients is provided in Fig. S1). We found that 94% of the variance in angiotensin II concentration was explained by the fixed effects of angiotensin I (${R}_{m}^{2}$=0.826), and the model could be improved only slightly by the addition of ACE and ACE2 (${R}_{m}^{2}$=0.882). The variance in angiotensin 1-7 concentration was partly explained by angiotensin I (${R}_{m}^{2}$=0.519), followed by ACE2 (${R}_{m}^{2}$=0.305), and their combination together with ACE explained more than 70% of its variance (${R}_{m}^{2}$=0.740). The variance of ALT-S was explained by ACE2 (${R}_{m}^{2}$=0.551), and the model could be improved by adding angiotensin I and ACE (${R}_{m}^{2}$=0.716). The ACE-S model, however, showed the poorest goodness of fit when only one of each of the covariates was used (angiotensin I: ${R}_{m}^{2}$=0.161; ACE: ${R}_{m}^{2}$=0.081; ACE2: ${R}_{m}^{2}$=0.188). Even the combined model still explained only ~ 40% of the variance of ACE-S (${R}_{m}^{2}$=0.478).

### Outcome

**Figure 7:**
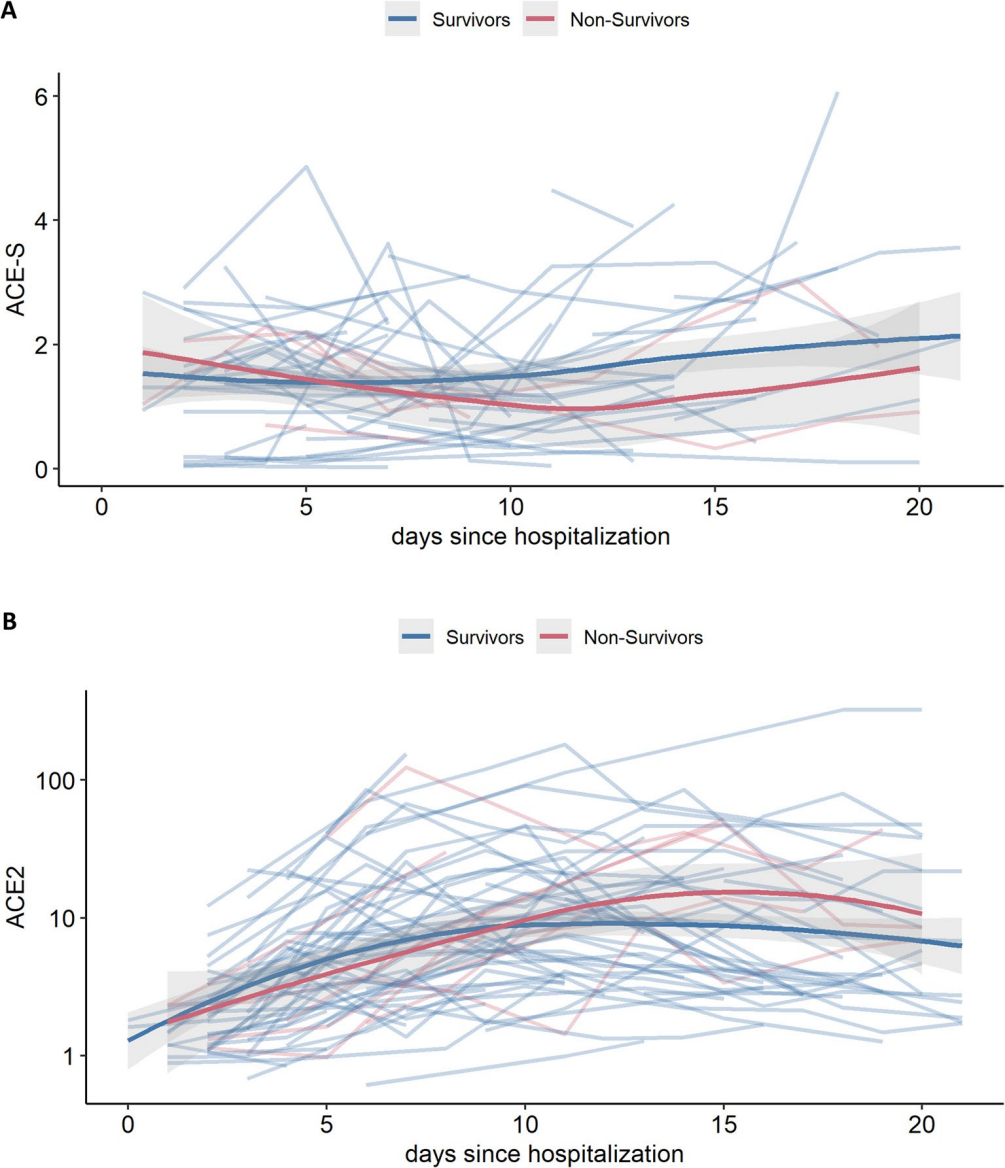
RAS and patient survival in severe COVID-19. ACE2 and ACE-S over time in severe COVID-19 patients stratified for patient survival.

Lastly, we compared trajectories of ACE2 and ACE-S in patients with severe COVID-19 with respect to outcome (Fig. 7, only severe patients included [n = 76]). However, despite the increase in ACE2 over time in severe COVID-19 patients (Fig. 3C), trajectories for ACE2 did not differ between survivors and non-survivors (Fig. 7A). Similarly, despite lower ACE-S in severe COVID-19 patients (Fig. 5A), trajectories for ACE-S did not differ between survivors and non-survivors (Fig. 7B).

## Discussion

In the present study, we analyzed systemic RAS in patients who were hospitalized with COVID-19. We predicted that the interaction between SARS-CoV-2 and ACE2 would result in systemic RAS dysregulation^4,6^. Our main findings were (i) that the observed increase in angiotensin II levels in severe COVID-19 was driven by an overall RAS activation and highest in mechanically ventilated patients and in those on RAS inhibitors; (ii) that the angiotensin II:I ratio as a marker for ACE activity (ACE-S) was reduced in severe COVID-19 and did not correlate with ACE concentration; (iii) that RAS was shifted to the alternative RAS axis with an increase in angiotensin 1-7 levels rather than angiotensin II; (iv) the shift to the alternative RAS axis was highly correlated with an increase in systemic ACE2 concentration; and (v) RAS profiles in most severe COVID-19 patients requiring mechanical ventilation resembled RAS profiles in severity of disease matched influenza patients.

The low ACE-S observed in severe COVID-19 (and influenza) could have resulted from decreased angiotensin II formation from angiotensin I or from increased enzymatic degradation of angiotensin II. ACE2 primarily mediates the latter, degrading angiotensin II to angiotensin 1-7 and counterbalancing the effects of angiotensin II. ACE2 has been described as a biomarker in various pathologic conditions, including hypertension and heart failure^22^, and occupies a central position in the pathophysiology of COVID-19.

Despite this association, some features of a proposed RAS dysregulation in COVID-19 suggested so far are hypothetical with respect to the human system: Kuba et al. used mouse models for their finding that SARS-CoV infection and the spike protein of SARS-CoV alone reduced ACE2 expression in the lungs^7^. Based on that study, many researchers suggested that in COVID-19, a lack of ACE2 activity following SARS-CoV-2 infection causes an increase in angiotensin II that might mediate lung injury. Of note, systemic RAS activity was not investigated in these studies, and the reported increase in local lung angiotensin II might in part have arisen from increased plasma renin activity, culminating in an increased rate of angiotensin II formation in the lungs. Previous studies have shown that in critically ill patients (including those with influenza), renin activity and angiotensin II levels correlate with tissue injury, disease severity, and outcome^23,24^. Early reports including RAS data from12 patients hospitalized for COVID-19 in Shenzhen (half of whom developed acute respiratory distress syndrome, or ARDS), showed that angiotensin II levels in severe COVID-19 were higher than in healthy controls and associated with viral load and lung injury^10^. We previously reported elevated angiotensin II levels in invasively ventilated COVID-19 patients in our cohort^11^.

In the present dataset, however, the angiotensin II elevation disappeared after we excluded patients on RAS inhibitors and was most likely a consequence of compensatory upregulation of renin in response to RAS blocker treatment rather than from the impaired degradation of angiotensin II by functional ACE2 deficiency. ARB and ACEi treatments are well known to cause increased renin activity^25^. We therefore performed all analysis stratified for concomitant RAS medication. These data underline the importance of accounting for RAS inhibitory medication in studies analyzing angiotensin profiles. The observed elevation in angiotensin II levels in critically ill patients may further be primarily attributable to hemodynamic instability and overall disease severity (including extra-pulmonary organ failure) rather than being a specific effect of SARS-CoV-2-mediated lung injury. In line with this reasoning, another study found no elevation in angiotensin levels in COVID-19 patients who were compared to “severity-of-disease” matched controls with respiratory failure^26^. In yet another study, the authors found that a decrease in serum angiotensin II levels in COVID-19 patients was correlated with subsequent lung damage^27^.

Remarkably, in the present study, systemic ACE2 in patients with severe COVID-19 increased to levels that profoundly affected systemic angiotensin metabolites, as shown by an increase in the ALT-S and in angiotensin 1-7 levels. The strong correlation between ACE2 and ALT-S provides evidence for unimpaired activity of the increased levels of soluble ACE2 (while it has been proposed that enzymatically inactive, truncated ACE2 variants are upregulated by inflammation) and for enzymatic cleavage of angiotensin II to angiotensin 1-7 primarily by ACE2 (and not by another unspecified peptidase)^28^. We have recently shown that ACE2 increases in bronchoalveolar lavage fluid following lipopolysaccharide instillation in a human endotoxemia model^29^. To our best understanding, our data both add to our previous findings^11^ and align perfectly with an autopsy study from Belgium showing increased pulmonary ACE2 expression and decreased ACE expression in ARDS with or without COVID-19, when compared with unaffected controls^30^. Of interest, as in our previous^11^ and present work, researchers in Canada reported an upward trajectory of soluble ACE2 at 7-day sampling (from baseline) that was independently associated with increased mortality risk among their 242 patients with COVID-19^31^. A relationship among ACE2, disease severity, and mortality emerged in yet another analysis, this one from Hungary, of 176 patients with COVID-19^32^, and five additional studies have demonstrated elevated to highly elevated ACE2 levels in COVID-19^33–37^. Three other studies found no ACE2 elevation in COVID-19^38–40^, possibly because the included patients were less severely affected and/or ACE2 was measured only at the beginning of the disease course.

Interestingly, ALT-S was also elevated in mechanically ventilated patients with influenza: We have previously also reported an increase in ACE2 in patients with influenza, although levels were lower compared to mechanically ventilated patients with COVID-19. Importantly, no longitudinal data were available for patients with influenza and changes over time could not be assessed.

The observed increase in ACE2 in COVID-19 patients, however, could only partly explain the observed reduction in ACE-S in severe COVID-19 that we identified in our descriptive mixed linear models. This result suggests that the relative decrease in angiotensin II levels is further influenced by an unaccounted enzymatic degradation of angiotensin II (in addition to the action of ACE2) to unmeasured downstream metabolites, or by the presence of an intrinsic functional ACEi. Mouse data showed that angiotensin II hydrolysis in the circulation primarily depends on alternative pathways such as prolyloligopeptidase (POP) compared to predominantly membrane-bound ACE2 and low circulating ACE2 levels in state of health^41^. However, one of the key observations of our study was that in patients with severe COVID-19 soluble ACE2 increases to such levels that directly impact on angiotensin 1-7 levels in the circulation reflecting an overall shift to alternative RAS metabolite levels. ACE-S was also reduced in our comparator group of patients with severe influenza. We note that reduced ACE activity has been described in patients with ARDS and sepsis and was associated with adverse outcome^42,43^. In ARDS patients the observed reduced ACE-S was also not associated with altered ACE protein levels^42^.

A major strength of the current work is the longitudinal assessment of changes in RAS metabolite concentrations in individual patients over the course of the disease. The shift to the alternative RAS axis was time dependent and increased with time, and the intrinsic ACE activity in severe COVID-19 was already low at hospitalization. A recent study showed that in patients who recovered from COVID-19, ACE2 levels remained elevated for up to 3 months following infection, suggesting that RAS dysregulation in COVID-19 may persist after the acute illness resolves^33^. However, our analysis is limited to key enzymatic pathways of the classical and alternative RAS. Although we identified ACE2 as an important mediator of reduced intrinsic ACE activity in severe COVID-19, a significant portion of this reduction remained unaccounted for, and alternative pathways of angiotensin II degradation or endogenous ACE inhibition should be sought in subsequent studies.

Limitations of our study are primarily owed to the observational study character that does not allow to infer causal relations of RAS dysregulation and disease severity. Recent experimental data suggest that the RAS represents a druggable target in severe lung injury^44^. Similar RAS profiles in patients with severe influenza suggest that changes may represent a more general response to severe lung injury or critical illness rather than COVID-19-specific alterations. The control group however lacks longitudinal data and warrants further research in RAS (dys)regulation in critically ill patients. Another limitation is that renin activity was not directly measured but only estimated as sum of key angiotensins (PRA-S). However, a strong linear correlation between renin and PRA-S has previously been reported^45^. ACE and ACE2 were both measured as enzyme-specific angiotensin formation rate. We used both citrate and heparin plasma to quantify ACE2 and ACE concentrations and citrate may have a significant impact on metalloprotease activities. We therefor performed validation experiments of matrix-specific effects of heparin and citrate plasma and did not find a difference (Table S9 and S10).

In conclusion, the current findings provide evidence for profound alteration in systemic RAS regulation in COVID-19. Specifically, in severe COVID-19, we observed a reduced angiotensin II:I ratio, used as a marker for functional ACE activity, a reduction that was partially explained by an overall shift to the alternative RAS axis because of increased ACE2 levels. These data add to our mechanistic understanding of COVID-19 and prompted us to hypothesize the existence of an unidentified enzymatic degradation of angiotensin II (in addition to ACE2) or the presence of an unidentified intrinsic functional inhibitor of ACE.

## Supplementary Information

Supplementary Information.

## Data Availability

Data are available from the authors upon reasonable request (roman.reindl-schwaighofer@meduniwien.ac.at).
