# Supplementary material for: The systemic renin-angiotensin system in COVID-19
Source: Sci Rep. 2022 Nov 22;12:20117. doi: 10.1038/s41598-022-24628-1 (PMC9684482; doi:10.1038/s41598-022-24628-1)

**Supplementary material**

**Figure S1. Mixed effects models (all patients included [also patients on RAS inhibitory medication])**

**
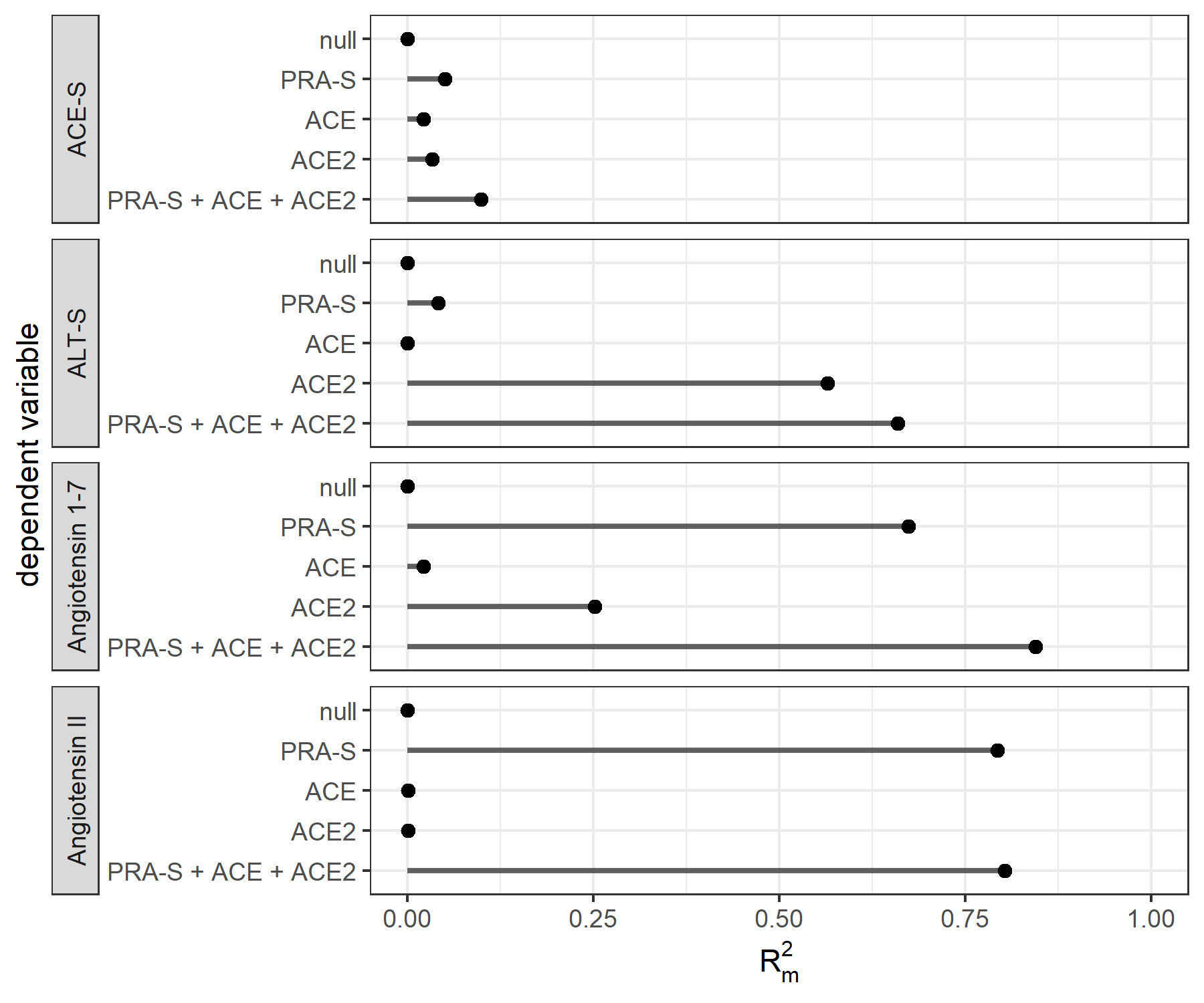
Legend:** $R_{m}^{2}$, i.e., marginal R squared for linear mixed effects regression, modeling angiotension 1-7, angiotension II, the angiotension II:I ratio (ACE-S), and alternative RAS ratio (ALT-S); all dependent variables were modeled using enzyme plasma renin activity (PRA-S) and enzyme concentration of ACE and ACE2 as single covariates, as well as with a combined model. All models included a random intercept per patient. $R_{m}^{2}$ denotes the variance explained by the fixed effects of the respective model. There was no correlation between ACE-S and RAS enzymes in patients on RAS inhibitors due to the effect of ACE inhibitors on ACE-S.

**Table S1. Results for the subpopulation not taking ACEi/ARBs.**

|  | **Non-severe (n=50)** | **Severe (n=42)** | **Total (N=92)** | **P** |
| --- | --- | --- | --- | --- |
| Age | 62 [44.7, 78.5] | 57 [46,73.5] | 62 [45.5, 76] | 0.888 |
| Female | 19 (40.4%) | 9 (22.0%) | 28 (31.8%) | 0.114 |
| BMI | 27.4 [23.7, 30.6] | 25.7 [24.4, 30.3] | 26.4 [24.2, 30.6] | 0.626 |
| Diabetes | 8 (16.0%) | 9 (21.4%) | 17 (18.5%) | 0.504 |
| Hypertension | 17 (34.0%) | 18 (42.9%) | 35 (38.0%) | 0.383 |
| COPD | 1 (2.0%) | 7 (16.7%) | 8 (8.7%) | 0.013 |
| Hospital length of stay (days) | 13.5 [9, 24] | 26.5 [16, 36] | 19.5 [12, 39\| | 0.003 |
| Death | 1 (2.0%) | 10 (23.8%) | 11 (12.0%) | 0.001 |

Continuous variables are presented as medians (with first and third quartiles; Q), and binary variables are presented as absolute numbers (with %).

**Table S2. 5-day intervals for the subpopulation not taking ACEi/ARBs.**

|  | **Time interval in days** | **Non-severe** | **Severe** | **P** |
| --- | --- | --- | --- | --- |
| PRA-S | (1–6) | 154.07 [29.46, 259.96] | 162.27 [29.18, 351.05] | 0.699 |
|  | (6–11) | 133.94 [58.04, 241.69] | 211.72 [79.10, 790.99] | 0.134 |
|  | (11–16) | 92.91 [48.51, 249.34] | 144.51 [51.23, 864.40] | 0.215 |
|  | (16–21) | 107.45 [59.59, 142.03] | 121.90 [37.39, 437.59] | 0.714 |
| Angiotensin I | (1–6) | 30.69 [4.55, 66.02] | 43.18 [11.21, 108.82] | 0.265 |
|  | (6–11) | 27.88 [12.48, 53.08] | 38.85 [20.61, 278.12] | 0.083 |
|  | (11–16) | 19.61 [9.33, 38.26] | 45.78 [10.68, 277.89] | 0.144 |
|  | (16–21) | 15.42 [8.46, 31.33] | 19.94 [5.24, 131.72] | 0.694 |
| Angiotensin II | (1–6) | 100.18 [17.95, 166.81] | 68.74 [15.06, 144.80] | 0.493 |
|  | (6–11) | 87.63 [42.80, 149.22] | 69.26 [32.66, 163.48] | 0.853 |
|  | (11–16) | 51.36 [29.02, 153.73] | 68.97 [23.37, 291.95] | 0.559 |
|  | (16–21) | 82.70 [44.09, 95.94] | 57.11 [26.33, 136.01] | 0.905 |
| Angiotensin 1-7 | (1–6) | 1.50 [1.50, 4.76] | 4.73 [1.50, 12.68] | 0.071 |
|  | (6–11) | 1.50 [1.50, 5.25] | 23.40 [4.33, 57.69] | <0.001* |
|  | (11–16) | 2.26 [1.50, 5.44] | 8.92 [3.51, 90.92] | <0.05* |
|  | (16–21) | 1.50 [1.50, 3.72] | 10.61 [1.50, 74.66] | 0.48 |
| Angiotensin 1-5 | (1–6) | 3.36 [1.00, 10.03] | 6.71 [1.14, 15.92] | 0.35 |
|  | (6–11) | 4.83 [3.53, 9.17] | 24.00 [5.11, 71.32] | <0.01* |
|  | (11–16) | 6.28 [3.91, 9.43] | 18.47 [8.92, 71.42] | <0.01* |
|  | (16–21) | 4.50 [2.75, 6.33] | 32.73 [4.95, 73.11] | 0.437 |
| ACE | (1–6) | 6.65 [5.14, 8.05] | 5.49 [4.74, 7.17] | 0.195 |
|  | (6–11) | 7.25 [6.04, 8.24] | 5.10 [4.22, 6.90] | <0.05* |
|  | (11–16) | 5.92 [4.83, 7.46] | 6.20 [4.42, 8.06] | 0.908 |
|  | (16–21) | 9.68 [7.68, 9.74] | 6.94 [5.03, 10.05] | 0.793 |
| ACE2 | (1–6) | 1.65 [1.02, 2.94] | 3.81 [1.65, 7.04] | <0.01* |
|  | (6–11) | 2.19 [1.37, 2.99] | 7.04 [5.02, 18.19] | <0.001* |
|  | (11–16) | 1.77 [1.29, 4.42] | 8.91 [3.95, 17.97] | <0.01* |
|  | (16–21) | 1.51 [1.24, 6.45] | 5.78 [2.92, 12.84] | 0.153 |
| ALT-S | (1–6) | 0.06 [0.04, 0.15] | 0.12 [0.06, 0.20] | <0.05* |
|  | (6–11) | 0.06 [0.05, 0.13] | 0.18 [0.13, 0.32] | <0.001* |
|  | (11–16) | 0.10 [0.07, 0.14] | 0.22 [0.12, 0.30] | <0.01* |
|  | (16–21) | 0.08 [0.07, 0.15] | 0.27 [0.12, 0.42] | 0.262 |
| ACE-S | (1–6) | 2.89 [2.21, 3.90] | 1.70 [1.01, 2.37] | <0.001* |
|  | (6–11) | 3.04 [2.25, 3.84] | 1.22 [0.82, 1.88] | <0.001* |
|  | (11–16) | 3.12 [2.31, 4.09] | 1.68 [1.05, 2.58] | <0.01* |
|  | (16–21) | 3.84 [3.07, 4.60] | 1.70 [0.89, 2.71] | 0.533 |

Values are presented as medians [with first, third quartiles]. Group comparisons (p values) were obtained using the Wilcoxon signed-rank test. All angiotensin concentrations, including PRA-S, are reported in pmol/L. ACE enzyme concentration is reported in µg/mL and ACE2 enzyme concentration in ng/mL. The ratio of angiotensin II to angiotensin I and the alternative RAS ratio are reported as unitless values.

**Table S3 – -day intervals for the subpopulation that was mechanically ventilated.**

|  | **Time interval in days** | **Mechanically ventilated, all** | **Mechanically ventilated, not taking ACEi/ARBs** |
| --- | --- | --- | --- |
| PRA-S | (1–6) | 390.81 [232.04, 1345.80] | 238.57 [158.96, 463.12] |
|  | (6–11) | 589.17 [243.26, 1022.16] | 406.22 [216.00, 889.76] |
|  | (11–16) | 96.94 [39.37, 807.20] | 96.94 [45.46, 793.55] |
|  | (16–21) | 136.02 [45.47, 465.98] | 163.77 [23.18, 528.87] |
| Angiotensin I | (1–6) | 190.52 [67.49, 584.65] | 100.68 [40.39, 285.39] |
|  | (6–11) | 239.99 [71.76, 502.61] | 166.02 [36.36, 410.69] |
|  | (11–16) | 24.08 [7.10, 271.23] | 24.08 [7.81, 259.07] |
|  | (16–21) | 28.14 [6.48, 146.26] | 23.41 [1.50, 167.82] |
| Angiotensin II | (1–6) | 148.19 [94.20, 498.04] | 98.26 [55.02, 217.90] |
|  | (6–11) | 167.96 [68.63, 396.64] | 85.46 [46.69, 252.45] |
|  | (11–16) | 48.10 [21.59, 272.13] | 48.10 [21.02, 158.64] |
|  | (16–21) | 57.43 [22.74, 136.21] | 59.71 [16.93, 161.45] |
| Angiotensin 1-7 | (1–6) | 15.49 [9.97, 54.17] | 11.09 [7.71, 29.86] |
|  | (6–11) | 69.94 [38.67, 165.16] | 56.34 [34.40, 110.35] |
|  | (11–16) | 8.53 [3.39, 112.17] | 8.53 [3.51, 90.92] |
|  | (16–21) | 5.42 [1.50, 74.66] | 19.71 [1.50, 92.98] |
| Angiotensin 1-5 | (1–6) | 17.24 [7.83, 62.87] | 16.53 [6.30, 33.73] |
|  | (6–11) | 75.14 [41.22, 159.20] | 69.44 [34.15, 83.88] |
|  | (11–16) | 16.32 [7.81, 124.60] | 16.32 [8.92, 71.42] |
|  | (16–21) | 12.56 [6.95, 73.11] | 58.68 [6.78, 77.92] |
| ACE | (1–6) | 5.73 [5.23, 7.86] | 5.49 [5.19, 7.35] |
|  | (6–11) | 5.35 [4.55, 7.13] | 4.85 [4.19, 6.78] |
|  | (11–16) | 6.70 [5.22, 8.44] | 6.73 [4.56, 8.46] |
|  | (16–21) | 7.04 [5.79, 9.80] | 7.33 [5.33, 10.40] |
| ACE2 | (1–6) | 4.50 [2.17, 8.92] | 4.42 [1.77, 9.61] |
|  | (6–11) | 11.57 [7.51, 24.71] | 9.53 [6.90, 23.50] |
|  | (11–16) | 12.60 [7.85, 30.37] | 11.84 [7.25, 18.70] |
|  | (16–21) | 7.92 [3.10, 19.77] | 7.60 [3.10, 14.46] |
| ALT-S | (1–6) | 0.12 [0.07, 0.17] | 0.12 [0.08, 0.16] |
|  | (6–11) | 0.27 [0.19, 0.38] | 0.30 [0.21, 0.38] |
|  | (11–16) | 0.26 [0.19, 0.32] | 0.26 [0.19, 0.30] |
|  | (16–21) | 0.19 [0.12, 0.42] | 0.37 [0.16, 0.44] |
| ACE-S | (1–6) | 1.41 [0.98, 1.84] | 1.70 [1.08, 2.05] |
|  | (6–11) | 0.98 [0.55, 1.41] | 1.07 [0.61, 1.32] |
|  | (11–16) | 1.36 [1.00, 2.08] | 1.36 [1.00, 2.22] |
|  | (16–21) | 1.93 [0.89, 2.78] | 0.91 [0.88, 1.70] |

Values are presented as medians [with first, third quartiles]. All angiotensin concentrations, including PRA-S, are reported in pmol/L. ACE enzyme concentration is reported in µg/mL and ACE2 enzyme concentration in ng/mL. The angiotensin II:I ratio and the alternative RAS ratio are reported as unitless values.

**Table S4: 5-day intervals for the subpopulation for the subgroup taking ACEi or ARB**

|  | **Time interval in days** | **ACEi** | **ACEi** | **ARB** | **ARB** |
| --- | --- | --- | --- | --- | --- |
|  |  | **non-severe** | **severe** | **non-severe** | **Severe** |
| PRA-S | [1,6] | 95.08 [39.36,548.59] | 612.19 [386.14,840.08] | 100.98 [52.68,127.10] | 416.80 [141.27,1634.05] |
|  | (6,11] | 83.00 [30.75,341.43] | 75.24 [37.90,776.75] | 55.46 [29.94,151.41] | 418.04 [246.75,853.55] |
|  | (11,16] | 50.37 [18.43,114.84] | 984.72 [619.10,1350.34] | 25.89 [16.97,34.81] | 40.76 [36.76,232.32] |
|  | (16,21] | 12.72 [12.72,12.72] | 229.96 [229.96,229.96] | NA [NA,NA] | 108.28 [88.26,277.34] |
| Angiotensin I | [1,6] | 86.03 [21.43,489.41] | 498.15 [278.48,576.89] | 15.79 [10.25,38.56] | 121.96 [41.91,576.52] |
|  | (6,11] | 54.93 [12.28,314.79] | 30.61 [9.86,360.81] | 10.75 [4.96,44.58] | 125.51 [75.40,266.64] |
|  | (11,16] | 39.51 [9.15,93.75] | 353.86 [207.44,500.28] | 4.22 [2.86,5.57] | 8.81 [6.51,64.79] |
|  | (16,21] | 6.97 [6.97,6.97] | 49.18 [49.18,49.18] | NA [NA,NA] | 32.87 [16.57,81.56] |
| Angiotensin II | [1,6] | 9.47 [4.75,34.20] | 101.08 [50.13,112.86] | 65.89 [34.44,79.31] | 251.87 [87.15,722.17] |
|  | (6,11] | 14.45 [5.77,25.89] | 32.93 [20.86,190.75] | 37.12 [19.54,87.42] | 171.75 [73.08,351.53] |
|  | (11,16] | 6.28 [5.27,7.37] | 464.60 [314.61,614.59] | 16.03 [8.91,23.14] | 21.85 [19.17,73.56] |
|  | (16,21] | 1.00 [1.00,1.00] | 158.73 [158.73,158.73] | NA [NA,NA] | 55.16 [34.20,60.49] |
| Angiotensin 1-5 | [1,6] | 1.00 [1.00,3.44] | 7.98 [6.42,17.78] | 4.36 [2.54,7.04] | 12.85 [4.27,210.98] |
|  | (6,11] | 1.00 [1.00,2.17] | 4.85 [1.51,24.50] | 3.51 [2.40,6.77] | 63.91 [14.93,148.71] |
|  | (11,16] | 1.00 [1.00,1.52] | 81.54 [47.13,115.95] | 1.89 [1.45,2.34] | 8.07 [6.95,49.45] |
|  | (16,21] | 1.00 [1.00,1.00] | 11.39 [11.39,11.39] | NA [NA,NA] | 10.56 [7.45,14.57] |
| Angiotensin 1-7 | [1,6] | 3.29 [1.50,15.25] | 30.43 [19.43,36.74] | 1.50 [1.50,5.01] | 10.10 [5.00,188.66] |
|  | (6,11] | 4.19 [1.50,12.62] | 4.59 [2.11,25.22] | 1.50 [1.50,3.48] | 48.74 [9.83,154.05] |
|  | (11,16] | 3.02 [1.50,10.57] | 77.32 [43.44,111.20] | 1.50 [1.50,1.50] | 2.60 [1.50,44.02] |
|  | (16,21] | 1.50 [1.50,1.50] | 4.93 [4.93,4.93] | NA [NA,NA] | 3.39 [1.50,7.44] |
| ACE | [1,6] | 6.07 [4.95,7.36] | 8.68 [7.25,9.09] | 5.77 [4.60,7.15] | 5.42 [4.25,5.81] |
|  | (6,11] | 5.33 [4.54,7.83] | 8.18 [7.70,8.75] | 6.23 [5.29,7.65] | 5.38 [4.87,5.67] |
|  | (11,16] | 8.33 [6.74,9.28] | 7.09 [6.49,8.48] | 8.13 [7.12,9.14] | 6.61 [5.77,6.93] |
|  | (16,21] | 4.21 [4.21,4.21] | 7.76 [7.50,9.42] | 7.15 [7.15,7.15] | 6.05 [5.84,6.91] |
| ACE2 | [1,6] | 1.80 [1.46,3.57] | 3.54 [2.65,4.78] | 2.13 [1.40,2.65] | 3.67 [1.97,10.06] |
|  | (6,11] | 4.27 [1.71,5.45] | 3.90 [2.95,11.38] | 2.53 [1.61,3.53] | 12.01 [8.77,38.10] |
|  | (11,16] | 3.87 [1.15,7.00] | 8.20 [3.31,16.25] | 2.91 [2.60,3.22] | 22.78 [7.82,33.66] |
|  | (16,21] | 3.75 [3.75,3.75] | 7.92 [3.74,19.37] | 2.26 [2.26,2.26] | 4.62 [2.41,13.04] |
| ACE-S | [1,6] | 0.11 [0.05,0.30] | 0.17 [0.15,0.88] | 2.91 [2.05,3.28] | 1.51 [1.31,1.67] |
|  | (6,11] | 0.18 [0.04,0.79] | 1.50 [1.27,1.58] | 2.35 [2.04,3.16] | 1.17 [0.60,1.48] |
|  | (11,16] | 0.09 [0.07,0.19] | 1.86 [1.50,2.22] | 4.37 [4.37,4.37] | 2.06 [1.78,2.39] |
|  | (16,21] | NA [NA,NA] | 3.23 [3.23,3.23] | NA [NA,NA] | 2.14 [1.72,3.65] |
| ALT-S | [1,6] | 0.06 [0.04,0.09] | 0.08 [0.07,0.10] | 0.08 [0.06,0.10] | 0.12 [0.05,0.20] |
|  | (6,11] | 0.08 [0.05,0.09] | 0.14 [0.09,0.18] | 0.09 [0.08,0.14] | 0.20 [0.13,0.34] |
|  | (11,16] | 0.14 [0.11,0.15] | 0.14 [0.12,0.16] | 0.21 [0.15,0.26] | 0.30 [0.20,0.39] |
|  | (16,21] | 0.20 [0.20,0.20] | 0.07 [0.07,0.07] | NA [NA,NA] | 0.17 [0.10,0.22] |

Values are presented as medians [with first, third quartiles]. All angiotensin concentrations, including PRA-S, are reported in pmol/L. ACE enzyme concentration is reported in µg/mL and ACE2 enzyme concentration in ng/mL. The angiotensin II:I ratio and the alternative RAS ratio are reported as unitless values.

**Table S5: RAS profile of healthy individuals and patients with COVID-19**

For COVID-19 patients, medians of patient averages are reported

|  | **healthy individuals** | **COVID-19 (all)** |
| --- | --- | --- |
| Angiotensin I | 35.7 (13.2,55.8) | 38.56 (10.14,120.23) |
| Angiotensin II | 136.9 (66.2,214.7) | 82.46 (20.20,170.24) |
| Angiotensin 1-5 | 5.6 (1.0,9.1) | 4.78 (1.95,16.67) |
| Angiotensin 1-7 | 1.5 (1.5,1.5) | 3.39 (1.50,11.04) |
| PRA-S | 195.6 (79.3,249.7) | 152.68 (44.97,342.06) |
| ACE2 | 1.4 (1.4,1.4) | 2.72 (1.68,6.08) |
| ALT-S | 0.04(0.03,0.05) | 0.09 (0.05,0.14) |
| ACE-S | 4.7 (2.97,6.45) | 2.19 (1.44,3.14) |

All healthy individuals were male and median age was 26 (25,33) years.

**Table S6: RAS profile of mechanically ventilated patients with COVID-19 and influenza.**

For COVID-19 patients, medians of patient averages are reported

|  | **mechanically ventilated COVID-19** | **mechanically ventilated influenza** |
| --- | --- | --- |
| Angiotensin I | 187.85 (27.08,356.45) | 136.3(33.4,205.3) |
| Angiotensin II | 137.1 (39.5,292.8) | 1.0 (1.0,7.7) |
| Angiotensin 1-5 | 79.34 (21.76,148.72) | 39.0 (5.6,137.6) |
| Angiotensin 1-7 | 55.77 (12.77,145.20) | 19.7(3.4,70.5) |
| PRA-S | 470.95 (138.48,922.47) | 393.4 (135.9,649.5) |
| ACE2 | 10.12 (5.14,17.71) | 3.4 (2.4,6.7) |
| ALT-S | 0.25 (0.21,0.34) | 0.14 (0.06,0.23) |
| ACE-S | 1.51 (0.90,2.03) | 1.49 (1.14,3.16) |

**Table S7: RAS for COVID-19 patients on ACEi, ARB or no RASi medication.**

|  | **No ACEi/ARB** | **ACEi** | **ARB** | **P** | |
| --- | --- | --- | --- | --- | --- |
|  |  |  |  | ACEi vs non | ARB vs non |
| Angiotensin I | 32.68 (8.03,67.41) | 144.26 (37.52,383.93) | 48.17 (10.71,122.96) | <0.01 | 0.132 |
| Angiotensin II | 91.97 (26.35,170.47) | 21.07 (6.83,65.97) | 87.15 (40.55,186.28) | <0.01 | 0.454 |
| Angiotensin 1-5 | 6.30 (2.03,13.78) | 2.33 (1.09,6.63) | 6.76 (2.95,35.05) | 0.103 | 0.185 |
| Angiotensin 1-7 | 2.63 (1.50,7.76) | 10.25 (2.20,18.11) | 5.80 (1.50,28.48) | <0.05 | 0.272 |
| PRA-S | 144.78 (40.41,267.49) | 263.45 (66.44,445.18) | 168.24 (59.15,380.65) | 0.234 | 0.295 |
| ACE | 6.60 (4.94,7.98) | 7.50 (5.18,8.84) | 5.96 (4.70,6.47) | 0.179 | 0.141 |
| ACE2 | 2.44 (1.39,5.32) | 3.86 (2.00,5.13) | 2.83 (1.94,7.89) | 0.216 | 0.125 |
| ALT-S | 0.09 (0.05,0.15) | 0.09 (0.05,0.10) | 0.09 (0.07,0.13) | 0.612 | 0.33 |
| ACE-S | 2.66 (1.89,3.59) | 0.43 (0.10,0.74) | 2.08 (1.51,2.47) | <0.001 | <0.05 |

Values are presented as medians [with first, third quartiles]. All angiotensin concentrations, including PRA-S, are reported in pmol/L. ACE enzyme concentration is reported in µg/mL and ACE2 enzyme concentration in ng/mL. The angiotensin II:I ratio and the alternative RAS ratio are reported as unitless values.

**Supplementary Methods**

Validation of matrix specific effects on enzymatic activity of ACE2.

**Table S9:** Ang 1-7 formation determined in Serum / Heparin Plasma / EDTA / Citrate plasma of five individuals +/- spiked 10 ng/m rhACE2. The delta (spiked – unspiked) was calculated. (Det = Determination)

| 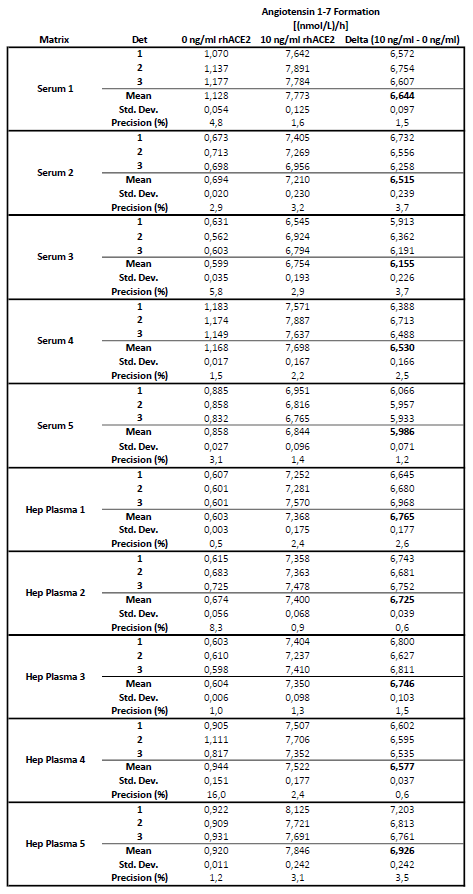 | 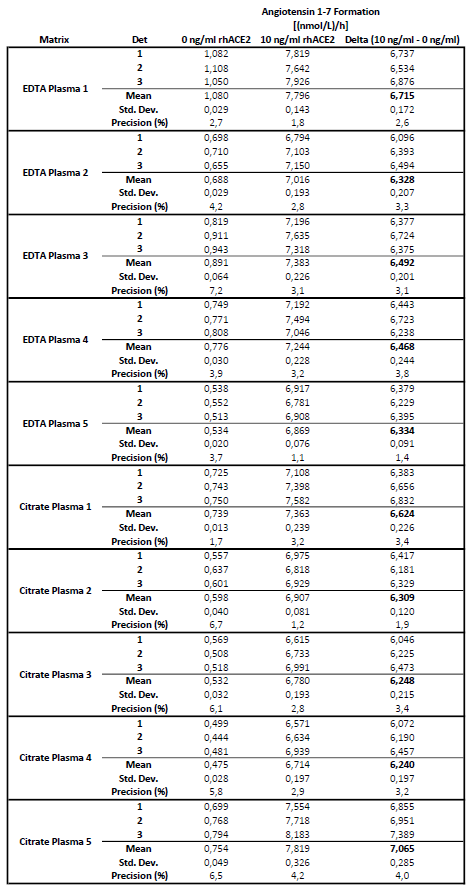 |
| --- | --- |

**Table S10:** Summary of Mean of Delta of Table 1 for Serum / Heparin / EDTA / Citrate plasma of all five individuals and the calculated mean thereof.


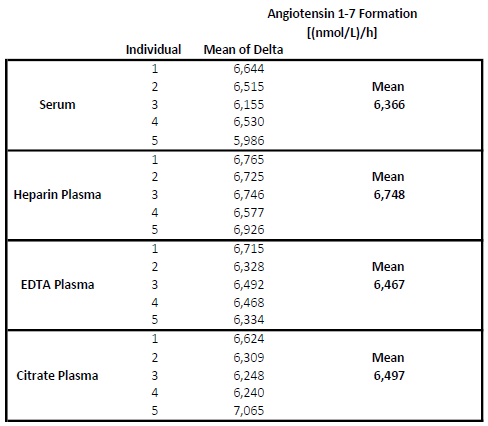

Supplement: Supplementary file 1 — Supplementary Information. [file 41598_2022_24628_MOESM1_ESM.docx]
